# Surface modification of PMMA polymer and its composites with PC_61_BM fullerene derivative using an atmospheric pressure microwave argon plasma sheet

**DOI:** 10.1038/s41598-021-88553-5

**Published:** 2021-04-29

**Authors:** Andrzej Sikora, Dariusz Czylkowski, Bartosz Hrycak, Magdalena Moczała-Dusanowska, Marcin Łapiński, Mirosław Dors, Mariusz Jasiński

**Affiliations:** 1 Wroclaw University of Science and Technology, Faculty of Electronics of Microsystems and Photonics, Janiszewskiego 11/17, 50-372 Wrocław, Poland; 2 Institute of Fluid Flow Machinery, Polish Academy of Sciences, Fiszera 14, 80-231 Gdańsk, Poland; 3 Division of Electrotechnology and Materials Science, Electrotechnical Institute, M. Skłodowskiej-Curie 55/61, 50-369 Wrocław, Poland; 4 Institute of Nanotechnology and Materials Engineering, Faculty of Applied Physics and Mathematics, Gdańsk University of Technology, Gabriela Narutowicza 11/12, 80-233 Gdańsk, Poland

**Keywords:** Materials science, Applied physics, Plasma physics

## Abstract

This paper presents the results of experimental investigations of the plasma surface modification of a poly(methyl methacrylate) (PMMA) polymer and PMMA composites with a [6,6]-phenyl-C61-butyric acid methyl ester fullerene derivative (PC_61_BM). An atmospheric pressure microwave (2.45 GHz) argon plasma sheet was used. The experimental parameters were: an argon (Ar) flow rate (up to 20 NL/min), microwave power (up to 530 W), number of plasma scans (up to 3) and, the kind of treated material. In order to assess the plasma effect, the possible changes in the wettability, roughness, chemical composition, and mechanical properties of the plasma-treated samples’ surfaces were evaluated by water contact angle goniometry (WCA), atomic force microscopy (AFM), attenuated total reflectance Fourier transform infrared spectroscopy (ATR-FTIR) and X-ray photoelectron spectroscopy (XPS). The best result concerning the water contact angle reduction was from 83° to 29.7° for the PMMA material. The ageing studies of the PMMA plasma-modified surface showed long term (100 h) improved wettability. As a result of plasma treating, changes in the samples surface roughness parameters were observed, however their dependence on the number of plasma scans is irregular. The ATR-FTIR spectra of the PMMA plasma-treated surfaces showed only slight changes in comparison with the spectra of an untreated sample. The more significant differences were demonstrated by XPS measurements indicating the surface chemical composition changes after plasma treatment and revealing the oxygen to carbon ratio increase from 0.1 to 0.4.

## Introduction

**Figure 1:**
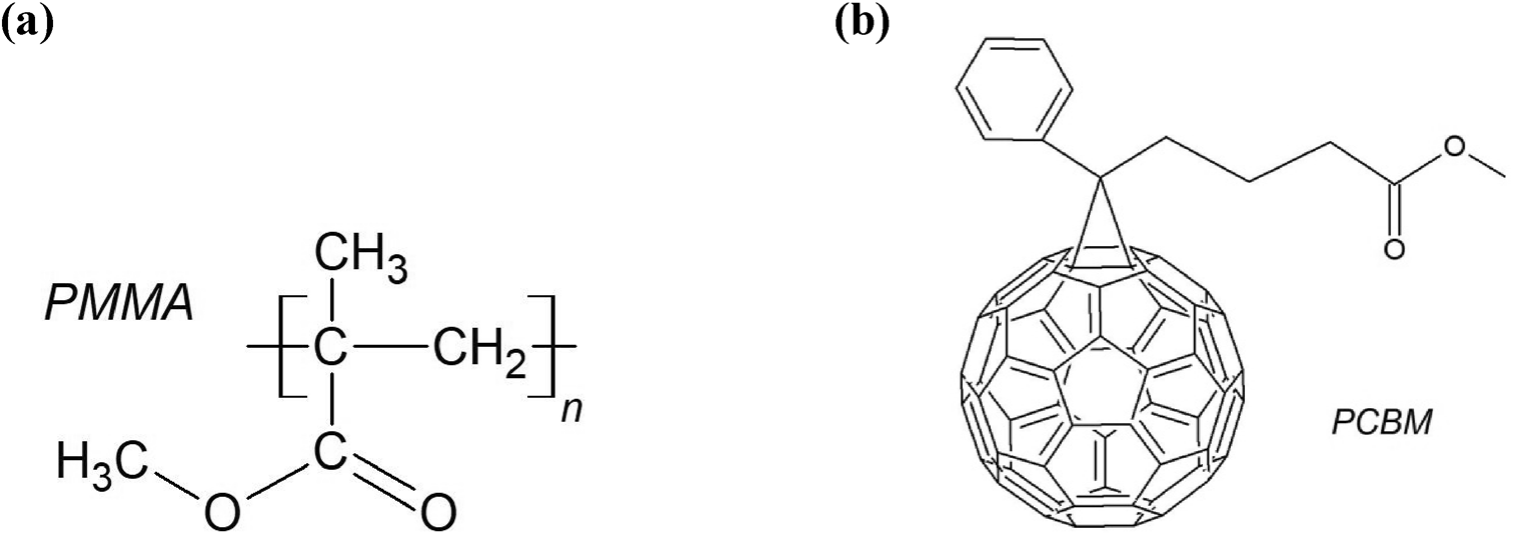
Chemical structures of (**a**) poly(methyl methacrylate) (PMMA) and (**b**) [6,6]-phenyl-C61-butyric acid methyl ester (PC_61_BM).

Poly(methyl methacrylate) (PMMA) of a chemical formula of (C_5_O_2_H_8_)_n_ is a synthetic acrylic polymer based on a methyl methacrylate monomer. The chemical structure of the PMMA polymer repeating unit is shown in Fig. 1a. Due to its unique properties, such as high transparency, biocompatibility with human tissue, and low cost of production, to list only a few, PMMA has found practical applications in various fields; not only in ophthalmology as an eye-glasses material or in biomedicine as a material for prosthetic implants but also as a high radiation indicator^1^.

A full review of the physical and chemical properties of PMMA and its applications can be found in the literature^2^. However, some possible applications of PMMA require higher surface energy, which similarly to other polymers, is low, which is the reason for the poor hydrophilicity and adhesion of the PMMA material’s surface. Thus, it is necessary to modify the hydrophilic-hydrophobic PMMA surface properties to obtain material of desired surface characteristics. There are a number of polymer material surface modification methods. These can be categorised as mechanical, chemical, and physicochemical methods^3^, or they can be divided into physical, chemical, thermal, and optical methods^4^. Obviously, each of these methods is accompanied by specific drawbacks, inconveniences and limitations.

In the last few years, a large number of studies have been performed aimed at improving the polymer’s surface properties by plasma treatment. The most important advantage of using gaseous plasmas to modify the material’s surface is that a plasma with suitably selected operational parameters is capable of changing the physical, chemical, and mechanical surface properties over a depth of only a few hundred Ångströms, leaving the base material bulk properties unchanged^5–9^. In the literature a number of review^9–13^ and report^14–24^ papers on the use of an atmospheric pressure plasma for surface modification of different polymers can be found. The plasma-based method was also applied in the case of the surface of the PMMA material^8,25–41^. However, in the case of PMMA, not only studies aimed at improving the PMMA surface properties have been performed, but also at improving its bulk properties. Namely, these investigations focussed on improving the thermal and/or thermo-oxidative stability of PMMA. PMMA is characterised by a relatively low thermal stability, and at high temperatures PMMA, is depolymerised by a radical chain reaction resulting with the formation of a mixture of polymer and methyl methacrylate (MMA) monomer^42^. As was shown, by compounding the PMMA polymer with C_60_ fullerene and its derivatives, this limitation can be overcome^34,42–45^. In this way, a material of new characteristics is formed. Improving and developing new technologies involving PMMA composites with fullerenes depends on the knowledge about their properties, including their surface properties after plasma treatment. Meanwhile, there is a lack of knowledge (or it is insufficient) about changes of the hydrophilic-hydrophobic and mechanical and morphological properties of samples of plasma-treated PMMA composites with fullerenes. Thus, it is not a well-known field.

With the issues raised in the introduction in mind, the aim of this paper is to present the experimental results of studies that focus on changes caused by the surface treatment of PMMA polymer and its composites with a PC_61_BM fullerene derivative. The chemical structure of PC_61_BM fullerene is shown in Fig. 1b. Among all of the different plasma sources, in the present work, we applied an atmospheric pressure argon microwave (2.45 GHz) plasma sheet. The use of this type of plasma is justified for several reasons. An atmospheric pressure plasma, in contrast to low pressure plasmas, offers reduced operational cost, and simplifies the set-up for plasma surface modification because, in this case, any vacuum apparatus (such as vacuum pumps, vacuum chambers, pressure meters, valves), are not involved. Since our plasma is driven by microwaves of a standard frequency of 2.45 GHz and with a microwave power not exceeding the 1000 W, a cheap commercial magnetron such as those commonly used in home microwave ovens, and standard waveguide components can be used. Another argument is the electrodeless operation, guaranteeing a high-purity plasma, free from any contamination from the electrode material. Argon as a plasma forming gas was used because of its low price (compared to other rare gases) and also because of argon plasma’s documented capability in the surface modification of various polymers (e.g. in the case of polycarbonate^46^, polyethylene terephthalate^47^, and polytetrafluoroethylene^48^). Finally, it should be also mentioned here that the unusual shape of the plasma used by us, which has the shape of a wide regular argon plasma sheet, provides the ability to process a large surface area at once. The purpose of this work is to assess the effect of the plasma on the wettability, roughness, chemical composition, and mechanical characteristics of the plasma treated samples’ surfaces by using water contact angle goniometry (WCA), atomic force microscopy (AFM), and attenuated total reflectance Fourier transform infrared spectroscopy (ATR-FTIR).

## Materials and methods

### Materials

In order to obtain the experimental specimens, four PMMA/PC_61_BM samples with the following mass proportions were prepared: 10%, 20%, 30%, and 40%. In addition, as a reference, a PMMA sample without PC_61_BM was prepared as well. For the fabrication process, PC_61_BM 99.5% (Solenne BV), chloroform HPLC (Sigma Aldrich), and PMMA (commercially available sheets) were used. Firstly, the PMMA was dissolved in chloroform. Next, the obtained solution was divided into vials, approx. 1 ml each. Then PC_61_BM was added to the vials and sonificated for 20 min. Finally, a dispersion of the PC_61_BM in the PMMA was spin-cast on a clear glass substrate at 1500 rpm for 25 s. The probe liquids for water contact angle measurement were distilled water from a local supplier. Argon of purity of ≥ 99.998 (% vol.), used as the plasma forming gas, was purchased from Air Liquide Polska.

### Atmospheric pressure plasma treatment on samples

**Figure 2:**
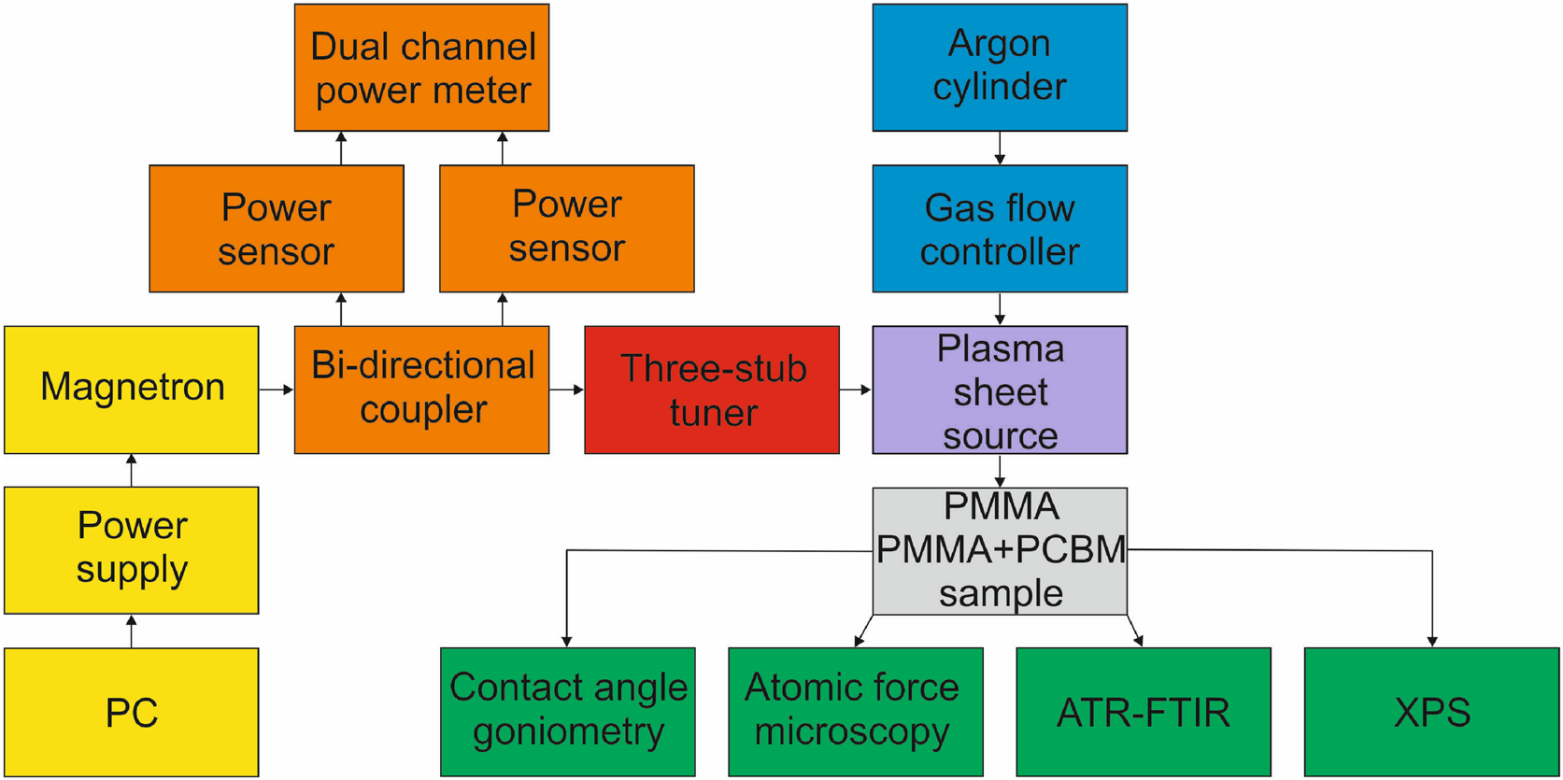
Schematic diagram of the experimental setup.

**Figure 3:**
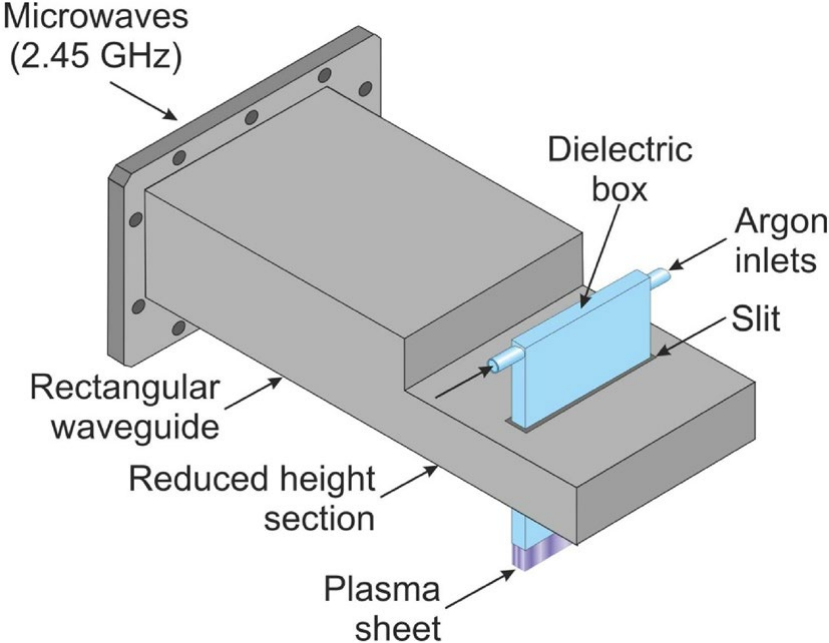
Schematic drawing of the microwave plasma sheet source.

Figure 2 shows the essential components of the experimental set-up for the generation of an atmospheric pressure microwave (2.45 GHz) argon plasma sheet. It consists of a plasma sheet source which was designed, built, tested, and patented^49^ by us and previously described^50–53^. Its schematic drawing is shown in Fig. 3. As can be seen, it has a waveguide-based structure with a reduced-height waveguide section with two rectangular coupling slits through which a flat dielectric box made of quartz is penetrated. The dimensions of the quartz box are: a length of 120 mm, a width of 50 mm, and a thickness of 7.2 mm, with a space width within the quartz box of 1.2 mm. Argon, as a plasma forming gas, is introduced into the dielectric box via two opposite inlets placed in its upper part, while the open, narrower side of the dielectric box placed in its lower part forms the rectangular gas outlet. The microwave power is fed to the plasma sheet source from a standard magnetron (CEFEMO 2M240H), like those usually used in home microwave ovens, operated at frequency of 2.45 GHz, and a maximum microwave power output of 1 kW. The magnetron is powered from a high voltage power supply made by Dipolar MagDrive (Sweden) and controlled using a personal computer with dedicated software. To improve the impedance matching in the waveguide system, thus to minimise the microwave reflected power, a three-stub tuner is placed between a bi-directional coupler and the plasma sheet source. The calibrated bi-directional coupler with two power sensors and dual channel power meter is used to monitor the incident and reflected microwave power levels. By subtracting the reflected microwave power from the incident microwave power, the absorbed microwave power is determined. The argon flow rate from the compressed argon cylinder is set by a Bronkhorst (Netherlands) EL-FLOW series mass gas flow controller.

**Figure 4:**
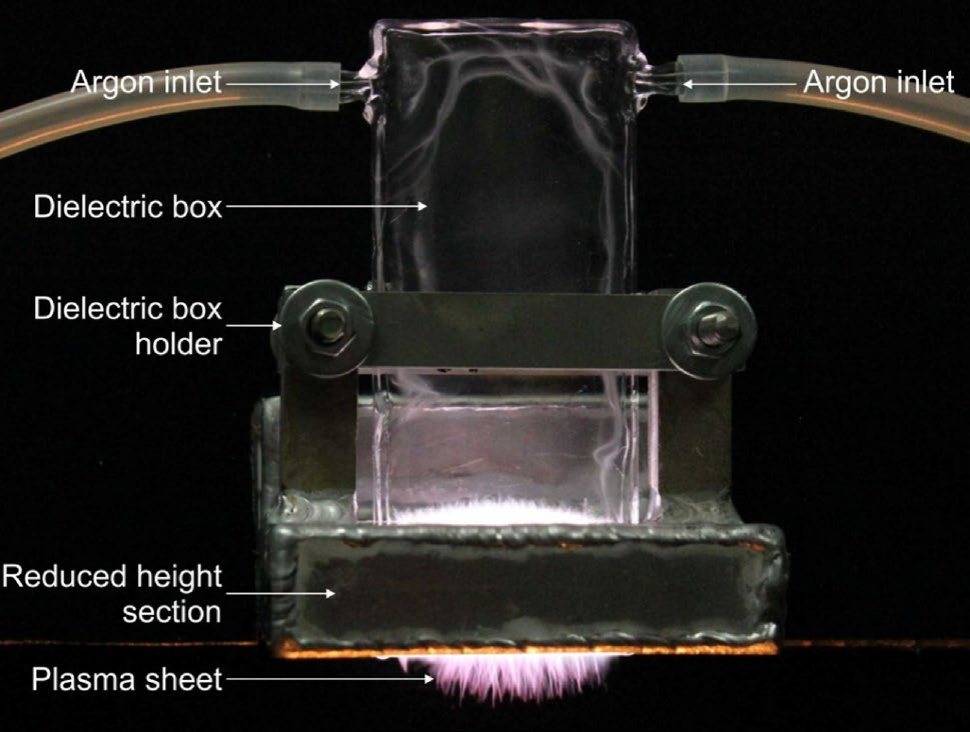
Photo of the microwave argon plasma sheet at argon flow rate of 20 NL/min and absorbed microwave power of 300 W.

In Fig. 4, a photo of the argon plasma sheet generated at a gas flow rate of 20 NL/min and microwave power delivered to the plasma of 300 W is shown. As can be seen in the figure, due to the argon gas flow, the generated plasma sheet comes out of the quartz box, enabling material surface treatment. The main merit of the plasma source presented herein is the shape of the obtained plasma, namely in the form of an argon plasma sheet formed by numerous argon filaments fluctuating within a limited quartz box volume, and therefore, its other benefit is its electrodeless operation. As it was shown in our previous paper^51^ due to the argon plasma filaments fluctuations, parameters of the plasma sheet outer part were the same at any axis position along the plasma sheet width. Such a unique and convenient plasma sheet shape allows large-area surface plasma treatment to be uniformly performed. Thus, the plasma sheet source described above was previously used by us for surface modification of polyethylene^54^ and polycarbonate^55^ materials.

In order to explore the application of the microwave argon plasma sheet described above in modifying the surface of PMMA polymers and PMMA + PC_61_BM composites, the samples were located on a motorised linear stage equipped with a stepper motor. The distance between the rectangular gas outlet of the quartz box and the linear stage was fixed to 20 mm. By applying the motorised linear stage, the tested samples were plasma treated with a constant velocity resulting from the linear stage movement velocity. The stage movement velocity thus the sample transfer velocity under the argon plasma sheet was adjusted to 10 mm/s. The tested samples were exposed downstream to the argon plasma under atmospheric pressure ambient air. The experimental tests were performed with an argon of a flow rate up to 20 NL/min and an microwave power up to 530 W. All of the experiment parameters mentioned above, including the sample distance from the plasma sheet, sample transfer velocity, argon flow rate, and microwave power were chosen to avoid thermal degradation of the surface treated samples.

### Water contact angle goniometry

The wettability changes of the plasma-treated samples’ surfaces was evaluated on the basis of the static water contact angle (WCA) measured according to standard water goniometry method. A lab-made experimental arrangement for WCA measurement was used, which was also applied for the analysis of ageing effect. The experimental arrangement consisted of a microcontroller-based water dispenser, a source of light, a microscope equipped with a CCD camera (Opticon, Wrocław, Poland), and a personal computer with a custom-written software installed. In the measurements, distilled water was used, with small drops being deposited dropwise on the surface of the tested samples. The water contact angle was determined by analysing two digital images from opposite sides of the distilled water drop. To fully evaluate the changes in the samples’ wettability, the WCA of the samples before and after treatment by plasma were compared. In order to ensure a valid statistical analysis, minimise statistical error, and ensure a certain level of reliability of the results for each tested sample, the WCA was determined from the measurements of at least eight drops of water placed on different parts of the sample surface. In the ageing effect study, the WCA was measured 2, 3, 4, 5, 24, and 100 h after the plasma modification. In every other case the WCA was determined immediately after the sample plasma treatment. The above mentioned immediate measurement was possible by arranging all experimental apparatus for the plasma processing and WCA measurement in a single laboratory room. All of the WCA tests were carried out under ambient conditions at the laboratory room of temperature of about 24 °C, and at a relative humidity of 35%.

### Atomic force microscopy (AFM)

To examine the microwave argon plasma sheet effect on the sample surface morphology and the sample surface mechanical properties, atomic force microscopy (AFM)^56^ studies were performed. The investigations were carried out using a DI3000 model of atomic force microscope from Digital Instruments (Santa Barbara, California, USA).

For the purposes of the topographical studies, the Tapping Mode^57^ was selected, which in contrast to contact mode, protects the samples’ surfaces against deterioration to a greater extent. A PointProbe (Nanosensors, Switzerland) with a tip radius r_tip*nominal*_ = 10 nm and a spring constant *k* = 43–68 N/m, and a resonance frequency *f*_res_ range from 306 to 353 kHz was used. For each sample surface, at least 10 scans were acquired. The examined surface area of scanning was 3 µm × 3 µm. The AFM images obtained were processed and then analysed using the Scanning Probe Image Processor (SPIP) processing software by Image Metrology A/S (Denmark)^58^. Finally, for each image, such surface roughness parameters as average roughness (Sa), root mean square roughness (S_q_), skewness (S_sk_), kurtosis (S_ku_), peak-peak value (S_z_), and surface area ratio (S_dr_) were determined. The definition of each of above mentioned parameters can be found, among others, in the literature^59–61^.

The changes in the samples’ surface mechanical properties were investigated by AFM in force spectroscopy mode^62^. Therefore, a CSG30 probe (NT-MDT Spectrum Instruments Ltd., Russia) was used in this mode. The probe’s nominal spring constant, *k*_*nominal*_, was 0.6 N/m. The real spring constant of the probe was determined using the thermal noise method^63^. In order to avoid the impact of the heterogeneity of the sample surface, and to provide representative data, measurements were taken at several points on each sample. The resulting data was processed and analysed using the SPIP software, which allowed mechanical parameters such as Young’s modulus, stiffness, transition indent, transition force, deformation and, adhesion force to be determined.

All of the AFM studies above described were processed in ambient air at a temperature of about 24 °C and at a relative humidity of 35%. In each case, AFM digital images were taken of the samples’ surfaces before and after being subjected to the microwave argon plasma sheet treatment. For each parameter, determined from the AFM images, the median and statistical errors were determined.

### Attenuated total reflectance Fourier transform infrared spectroscopy (ATR-FTIR)

The changes in the PMMA surface chemistry after plasma modification were investigated using attenuated total reflectance Fourier transform infrared spectroscopy (ATR-FTIR). For this purpose, a Thermo Scientific Nicolet 380 FTIR Spectrometer equipped with a PIKE Technologies GladiATR single reflection ATR accessory was used. The monolithic diamond ATR crystal was applied with a refractive index of 2.4 and a nominal angle of incidence of 45°. The ATR-FTIR spectra of the untreated and plasma-treated PMMA samples were recorded in the wavelength range of 4000 and 400 cm^−1^ at 4 cm^−1^ spectral resolution. The ATR-FTIR spectra of the plasma-modified sample were recorded immediately after plasma treatment. The ATR-FTIR background signal was measured in ambient air.

### X-ray photoelectron spectroscopy (XPS)

The more detailed chemical composition of samples surface was analyzed by X-ray photoemission spectroscopy (XPS). XPS measurement was carried out using Omicron NanoScience equipment. Samples were measured at room temperature, under the ultra-high vacuum conditions and pressures below 1.1 × 10^–8^ mBar. The photoelectrons were excited by a Mg-Kα X-ray source operated at 15 keV and 300 W. Argus hemispherical spectrophotometer equipped with 128 channel detector was used for photoelectrons energy measurements. The data analysis was performed with the CASA XPS software, using Shirley background subtraction and Gauss-Lorentz curve fitting algorithm by the least-squares method—GL (30).

## Results and discussion

### Surface wettability and ageing

**Figure 5:**
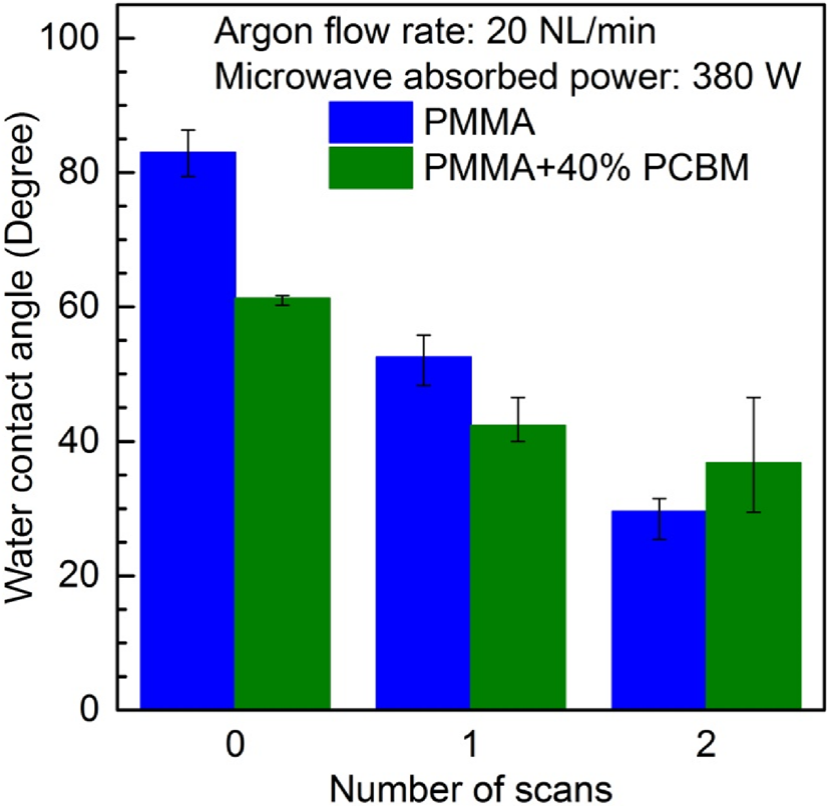
Effect of the plasma surface modification of PMMA polymer and PMMA + 40% PC_61_BM composite on the water contact angle as a function of the number of plasma scans. Argon flow rate 20 NL/min. Microwave power 380 W.

The relationship between the WCA and the plasma scans number is shown in Fig. 5. Two series of measurements are presented. The first corresponds to PMMA polymer and the second to PMMA + PC_61_BM composite with mass proportions of 40%. The results presented were carried out for an Ar flow rate of 20 NL/min and microwave power absorbed in the plasma of 380 W. The number of scans from 0 to 2 with constant treatment velocity was performed. The number of scans equal to 0 corresponds to the case of the plasma-untreated samples. From the figure, it can be clearly seen that the WCA depends on the plasma scans number. An increase in the plasma scans number leads to a decrease in the WCA for both kind of samples. Thus, it is a plasma modification time-dependant effect. The water contact angle of the untreated PMMA polymer and PMMA + 40% PC_61_BM composite is equal to 83° and 61°, respectively. After a single plasma scan, the corresponding values drop to 52.5° and 42.4°. Increasing the number of plasma scans to two resulted in a further decrease in these values to 29.7° and 36.8°, respectively. One can conclude that a further increase in the number of plasma scans will lead to a further reduction of the WCA. However, it should be noted that this plasma-based process depends not only on the time, but also on the microwave power delivered to the plasma^55^. Increasing the number of plasma scans can cause a thermal effect resulting in the degradation of the surface morphology.

**Figure 6:**
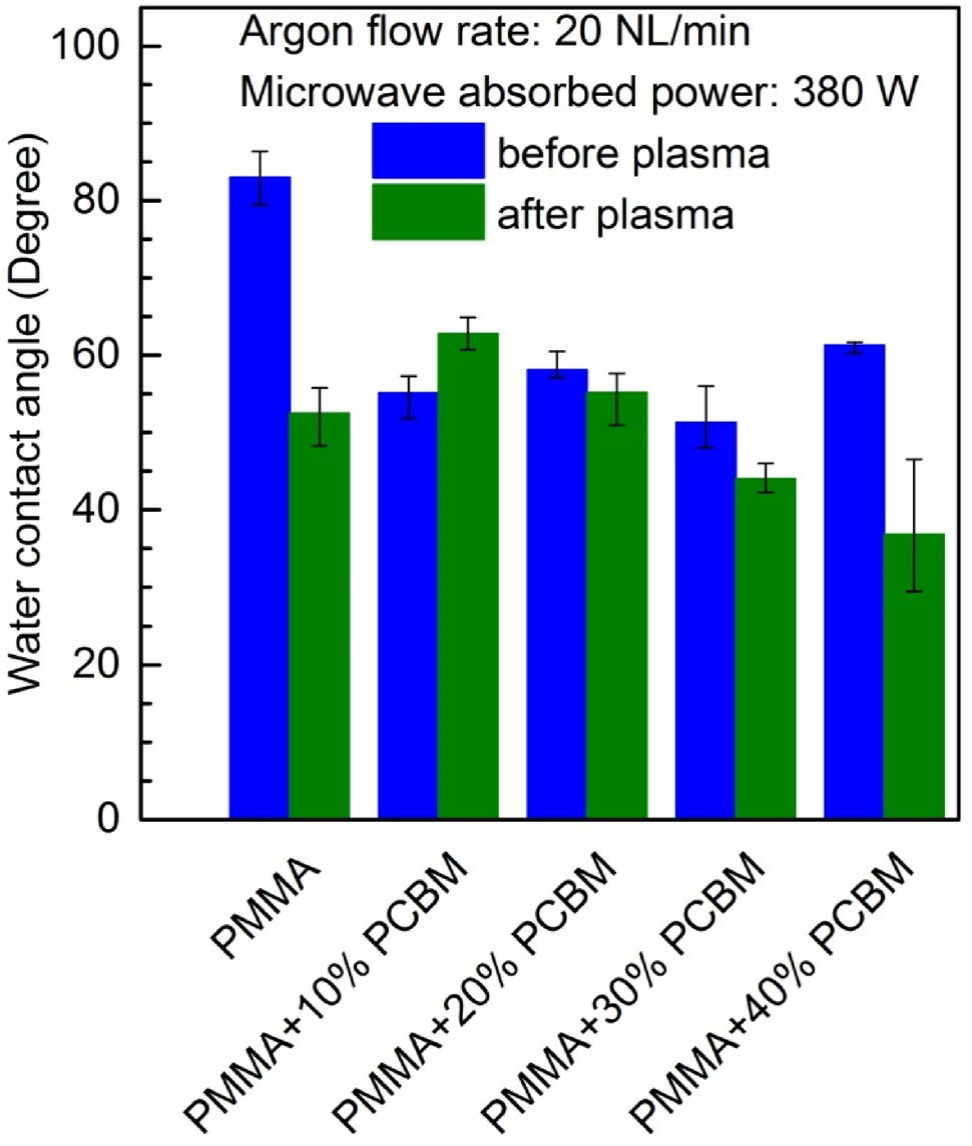
Effect of the plasma surface modification of PMMA polymer and PMMA + PC_61_BM composites on the water contact angle. Number of scans: 1. Argon flow rate 20 NL/min. Microwave power 380 W.

Figure 6 illustrates the effect of the plasma surface modification of PMMA polymer and PMMA + PC_61_BM composites on the water contact angle. Similarly, like in the case of the experimental results presented in Fig. 5, the results discussed corresponded to an Ar flow rate equal to 20 NL/min and absorbed microwave power equal to 380 W. However, in this case, the results concerning a single plasma scan of PMMA polymer and PMMA + PC_61_BM composite over the whole composition range are shown. In the figure, the blue bars indicate the results for the plasma-untreated samples. The green bars indicate the results after plasma treating. Generally, as can be seen from the figure, for almost all of the argon plasma sheet treated samples, a reduction in the WCA can be seen, however this is not the case for the PMMA + 10% PC_61_BM composite, for which the WCA before plasma treatment exhibited a lower value. Assuming the uniformity of the PMMA + PC_61_BM composites assured by process of their fabrication and the fact that for each tested sample, the WCA was determined from the measurements of a few drops of water placed on different parts of the sample surface, it may be concluded that the heterogeneity of the sample surface can’t be considered as is responsible for the reverse effect in the case of PMMA + 10% PC_61_BM composite. This behaviour of the sample is related due to the modification of the surface energy and roughness of the sample. A more significant reduction in WCA after plasma treatment can be observed in the case of the PMMA polymer, and the PMMA + 40% PC_61_BM composite, while in the case of the PMMA + 20% PC_61_BM, and PMMA + 30% PC_61_BM composites, only small changes in the WCA are seen.

It is worth of recalling here that, the above description refers to the static WCA measured by sessile-drop technique. Meanwhile, advancing and receding contact angles affords more valuable view on the hydrophilic-hydrophobic surface properties^64,65^. In the case of plasma-based polymer surface modification advancing and receding angles are more sensitive to the low- and high-energy components of the surface, respectively^64^. However, as we believe, also the investigation methodology used by us provides valuable data to be analysed.

**Figure 7:**
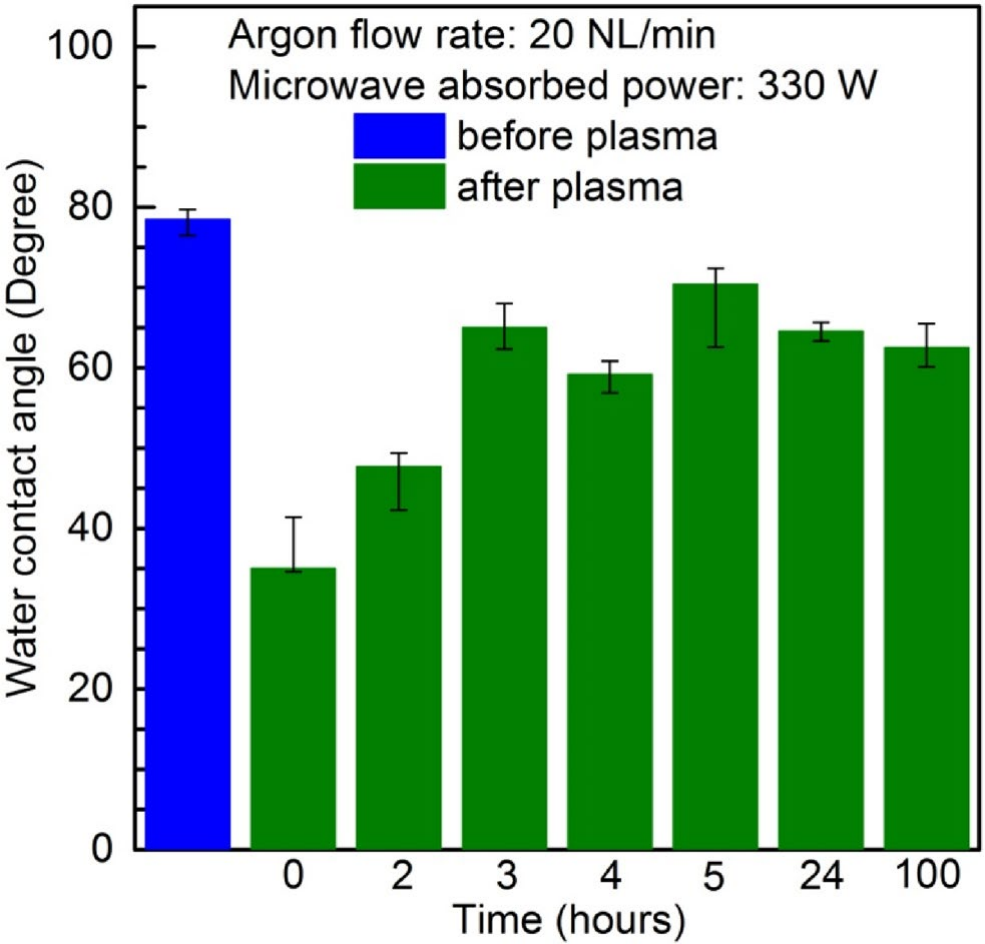
Water contact angle of the PMMA polymer surface as a function of time after plasma treatment. Argon flow rate 20 NL/min. Microwave power 330 W.

As is known, the main drawback of plasma surface modification is ageing, which means that the water contact angle of the plasma-treated polymeric surface is not stable over time, and increases depending on the sample store time^28,36^. To illustrate those phenomena, Fig. 7 shows the changes in the WCA as a function of time after plasma modification. The results refer to the PMMA polymer single treated by the argon plasma sheet at an Ar flow rate of 20 NL/min and microwave power delivered to the plasma of 330 W. In the figure, the results corresponding to the time of 0 h indicate the water contact measurement immediately after the plasma treatment. A general conclusion can be drawn from the Fig. 7 that the water contact angle increases with the time, but even after a period of 100 h after plasma treatment, does not reach the initial value of the water contact angle of a pristine sample, which was 78.5°. The water contact angle of the PMMA polymer after 100 h after plasma treatment was equal to 62.6° in this case.

It can be found in the literature that the so-called aging phenomenon of plasma-treated polymer surfaces is due to the instability of the polar hydrophilic groups^66^. During the storage time, the chemical polar groups change the orientation into the bulk of polymeric material. As a result of this, the chemical composition of the surface is changed by reducing the percentage of oxygen content and a decrease in the surface energy is also observed^31,67^. Thus, it should be concluded that the WCA increase with storage time is correlated only to the decrease in the surface energy, because the changes in the surface roughness should not reveal any reversible effect. Also, as is known, the ageing effect is not only time-dependent process but also depends on storage conditions like ambient environment and temperature^28,31,66–68^.

As was mentioned in the Introduction section, the hydrophilic properties of a PMMA surface can be modified using various types of plasma surface treatment techniques. In Table 1, a comparison of the recorded parameters of the WCA for different plasma-based techniques of PMMA polymer surface modification is presented. The responsible parameters and the results for the present research are given in the last row of the table. As can be seen in the Table 1, different types of plasmas, from the point of view of the method of plasma generation, the kind of plasma-forming gas, and the pressure have been taken into account. For instance, such kinds of plasmas as DC, AC, DBD, RF, and microwave are considered. Considering the plasma-forming gas, the table includes plasmas generated in rare gas (Ar), in molecular gases (O_2_, N_2_, air) and in gas mixtures (He + N_2_, He + CO_2_, He + O_2_,). The results for low and high pressure plasmas are also included in the table. As can be deduced from the table, the microwave argon plasma method of surface modification of PMMA polymer presented in this paper is comparable with other plasma-based methods, and in some cases is even competitive with other methods. In this respect, it is worth recalling here that our plasma source is operated under atmospheric pressure, and thus does not need a vacuum apparatus, making the plasma system for the material surface treatment simpler and cheaper. Worth mentioning the fact that, thanks to the unusual shape of the applied plasma in the form of a regular sheet, it is possible to treat large surface areas, thus also eliminating time-consuming and costly multiple scans of the surface to cover its entire area.

**Table 1:** Comparison of the plasma-based techniques for hydrophilicity improvement of PMMA polymer surface.

Plasma type	Plasma gas	Pressure	WCA of pristine sample	WCA after plasma treatment	References
RF	O_2_	75 Pa	83°	45°	^28^
DC	O_2_	0.2 Torr	72.5°	51°	^8^
RF	O_2_	100 bar	70.5°	26.1°	^32^
RF	O_2_	0.2 Torr	68.4°	24.6°	^33^
DBD	O_2_	Atm	85°	51°	^41^
RF	N_2_	0.5 Torr	76.3°	21.2°	^35^
RF	N_2_	0.5 Torr	76.3°	40.4°	^35^
DC	N_2_	3 × 10^–3^ Torr	63.84°	15.39°	^40^
DBD	N_2_	Atm	85°	44°	^41^
RF	Air	0.2 mbar	77.64°	61.31°	^26^
DBD	Air	Atm	85°	50°	^41^
Micro-barrier discharge	He + N_2_	Atm	80.1°	66.4°	^39^
Micro-barrier discharge	He + CO_2_	Atm	80.1°	56.9°	^39^
Micro-barrier discharge	He + O_2_	Atm	80.1°	70.5°	^39^
RF	He + O_2_	Atm	74°	40°	^25^
AC	He	Atm	69°	27°	^31^
AC	Ar + H_2_O	Atm	74°	43°	^36^
RF	Ar + H_2_O	Atm	80°	35°	^27^
RF	Ar	Atm	80°	20°	^27^
AC	Ar	Atm	69°	32°	^31^
Microwave	Ar	Atm	83°	29.7°	Present study

### Surface roughness

**Figure 8:**
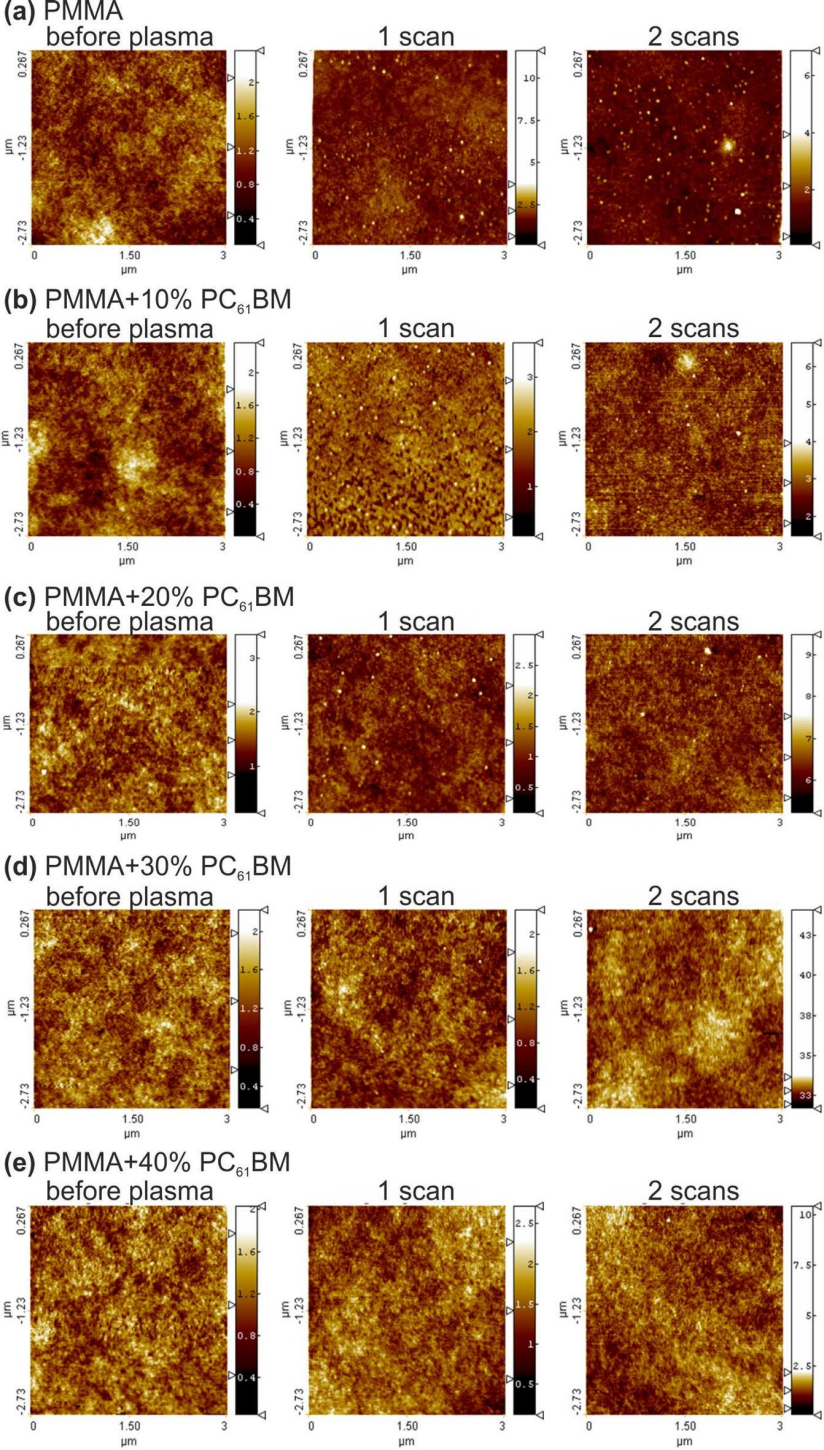
AFM acquired images of untreated and plasma treated PMMA polymer and PMMA + PC_61_BM composites. Argon flow rate 16 NL/min. Microwave power 330 W.

Exemplary AFM images acquired of untreated and microwave argon plasma-treated PMMA polymer and PMMA + PC_61_BM composites are shown in Fig. 8. The digital images of the plasma-treated samples correspond to an argon flow rate of 16 NL/min and absorbed microwave power of 330 W. The presented images illustrating the changes of the samples’ topography resulting from the plasma treatment are shown depending on the plasma scans number.

**Figure 9:**
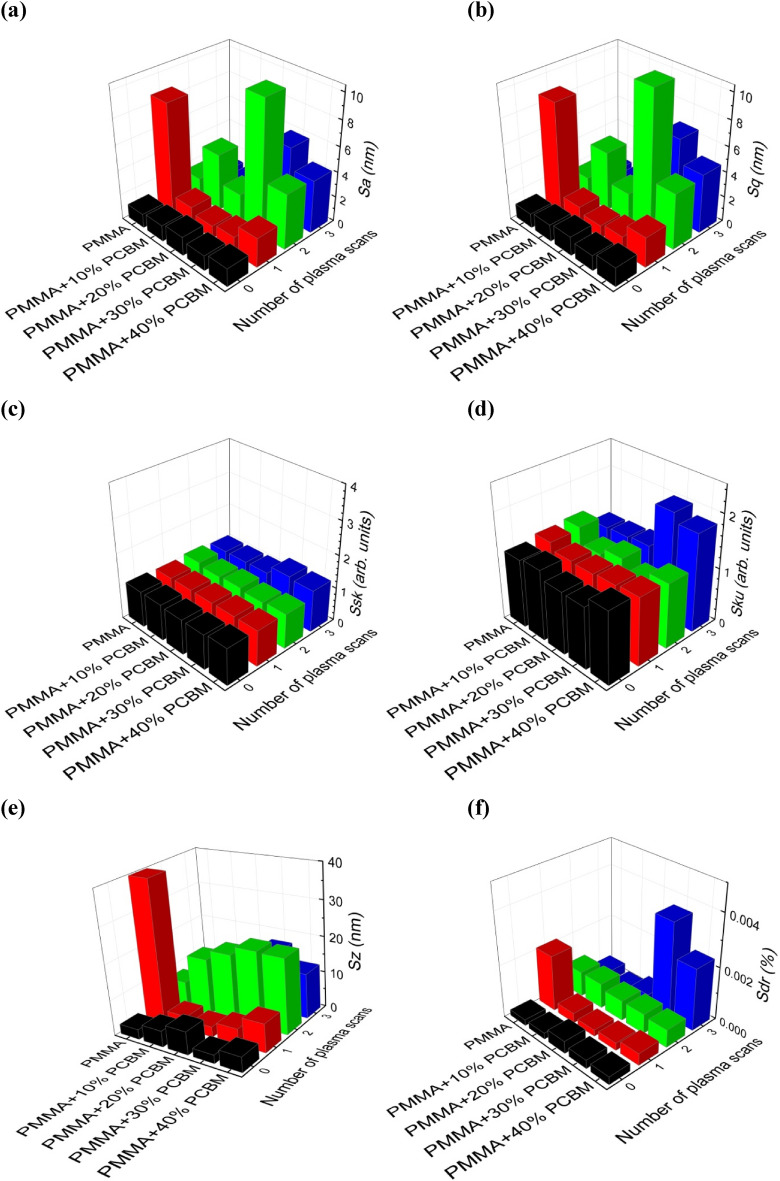
Effect of the plasma surface modification of PMMA polymer and PMMA + PC_61_BM composites on the surface roughness parameters: (**a**) S_a_; (**b**) S_q_; (**c**) S_sk_; (**d**) S_ku_; (**e**) S_z_; (**f**) S_dr_. Argon flow rate 20 NL/min. Microwave power 530 W.

As was mentioned in the “Materials and methods” section, the values of the surface roughness parameters were determined from the digital analysis of the AFM topography images. As a consequence of this, in Fig. 9, the 3D representation of average roughness (S_a_), root mean square roughness (S_q_), skewness (S_sk_), kurtosis (S_ku_), peak–peak value (S_z_), and surface area ratio (S_dr_) is presented as a function of the number of plasma scans for PMMA polymer and PMMA + PC_61_BM composites. The presented results refer to the argon plasma treatment at a gas flow rate of 20 NL/min and absorbed microwave power of 530 W.

As can be seen from Fig. 9a–f, the changes in the individual roughness parameters with the increase in the plasma scans number is nonlinear and irregular, so it is difficult to even formulate some general trends in this case. This applies to both the PMMA polymer, as well as the PMMA + PC_61_BM composites. For example, the root mean square roughness of the PMMA material after a single argon plasma scan increased from 1.1 nm for the PMMA pristine sample to 8.8 nm. Then, with the number of plasma scans increased to two, the root mean square roughness reached the value of 1.7 nm, and finally after three scans was equal to 1.1 nm. At the same time, in the case of PMMA + 30% PC_61_BM composite, the root mean square roughness was equal to 1.3 nm after a single plasma scan, after a second scan, was equal to 11.1 nm, but after three scans, was equal to 6.5 nm. A similar observation of the surface roughness behaviour with increasing the plasma scans number was also reported^25^ for the case of PMMA surface modification using an atmospheric pressure RF oxygen plasma. There, the root mean square roughness of a PMMA material first increased, then decreased, and finally increased again for the plasma scans number of 80, 144, and 208, respectively.

### Surface chemical composition

**Figure 10:**
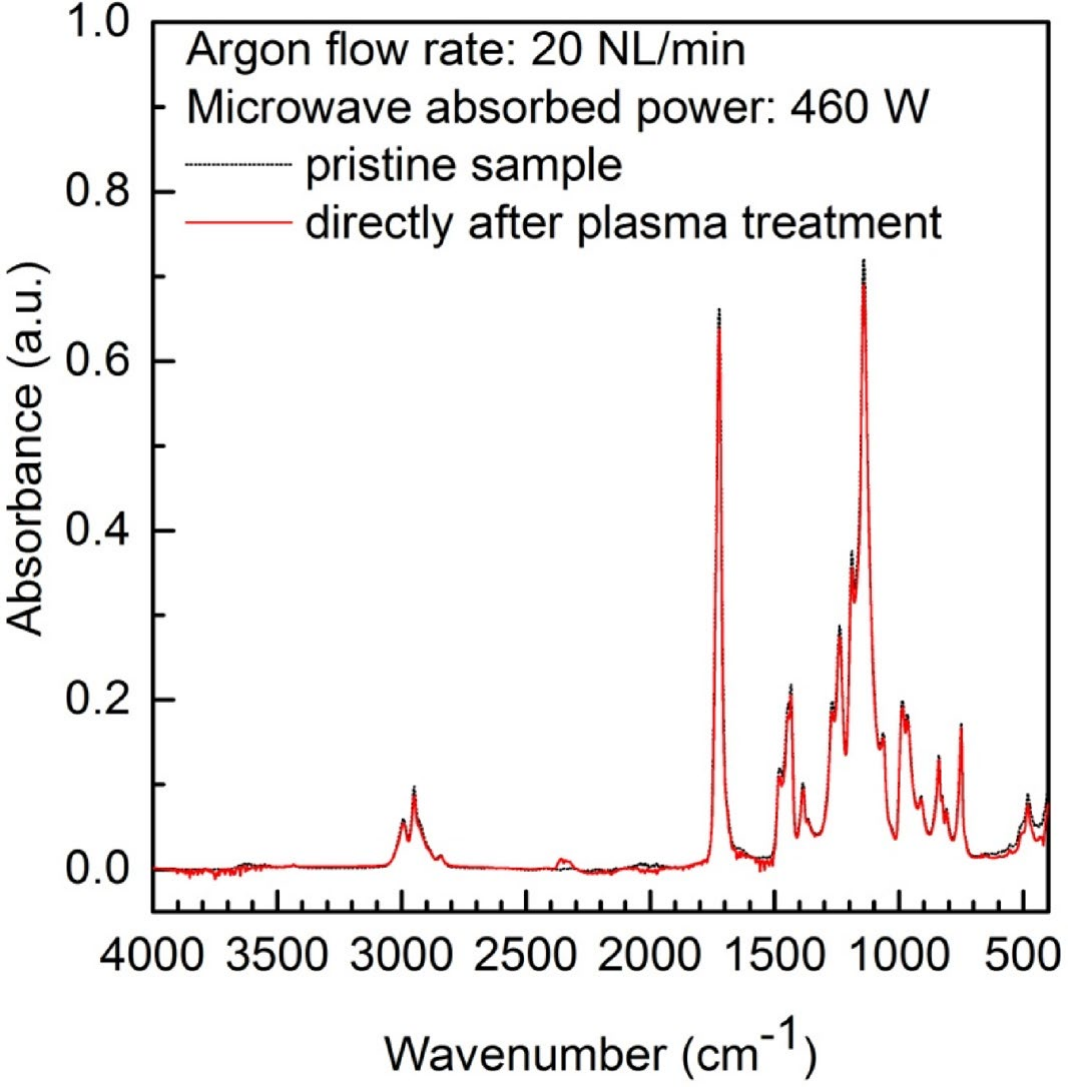
ATR FTIR spectra of PMMA polymer before and after plasma treatment measured in a wavenumber interval of 4000–400 cm^−1^. Argon flow rate 20 NL/min. Microwave power 460 W.

As was mentioned earlier, to study the changes in the microwave argon plasma-treated surface chemical composition of the PMMA samples, ATR-FTIR measurements were carried out. The ATR-FTIR surface spectra of a PMMA pristine sample and an argon plasma-modified PMMA sample recorded in a wavenumber interval of 4000–400 cm^−1^ for an argon flow rate of 20 NL/min and absorbed microwave power of 460 W are shown in Fig. 10.

The obtained measured ATR-FTIR spectrum of the PMMA pristine sample is in good agreement with the results of other experimental studies^25–27,30,33,35,40^. As can be seen from the figure, the ATR-FTIR spectra of the PMMA sample after argon plasma treatment does not reveal any significant variation. The ATR-FTIR spectra of the PMMA samples before and after plasma treatment indicate the same specific peaks of functional groups typical for a PMMA surface layer. A similar observation was noted in the case of low pressure plasma treatment of PMMA material in a humid argon microwave (2.45 GHz) plasma where no shift or change of single vibration modes in the ATR-FTIR spectra was detected^30^. Therefore, it may be concluded that as a result of the microwave argon plasma sheet treatment of the PMMA sample surfaces, no new functional groups are generated.

**Figure 11:**
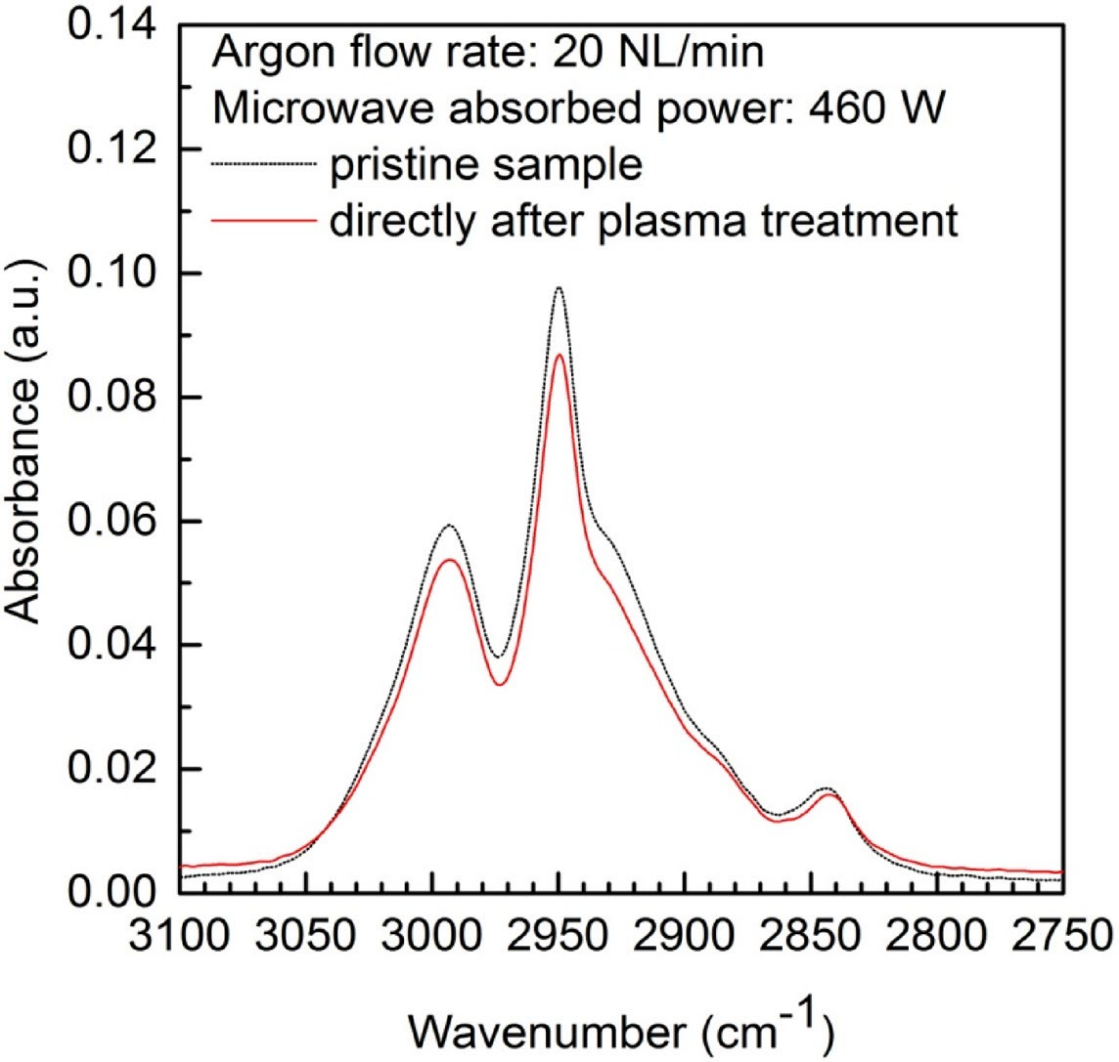
ATR FTIR spectra of PMMA polymer before and after plasma treatment in the wavenumber range of 3000–2750 cm^−1^. Argon flow rate 20 NL/min. Microwave power 460 W.

It was mentioned above that the ATR-FTIR spectra of the plasma-treated PMMA sample does not exhibit any significant differences in comparison with the spectra of a pristine PMMA sample, however small differences can be seen in certain spectral regions. These differences concern the changes in the intensity of individual spectral peaks. After a closer look, some differences of the peaks’ intensities can be seen in Fig. 10. This is particularly the case for the most intense ATR-FTIR spectral lines occurring at the wavenumbers of about 1722 cm^−1^ and about 1143 cm^−1^. These lines are attributed to the C=O stretching vibrations and C–O stretching vibrations, respectively. Both of these chemical polar bonds are connected with the wettability improvement, and are also responsible for the surface energy increase^40^. Figure 11 presents the ATR-FTIR spectra of the pristine and argon plasma-treated PMMA samples measured in the wavenumber range of 3000–2750 cm^−1^. In the figure, the changes in the peaks’ intensities corresponding to the C–H stretching vibration modes (occurring at 2993 cm^−1^ and 2950 cm^−1^) after argon plasma treatment can be clearly seen.

**Figure 12:**
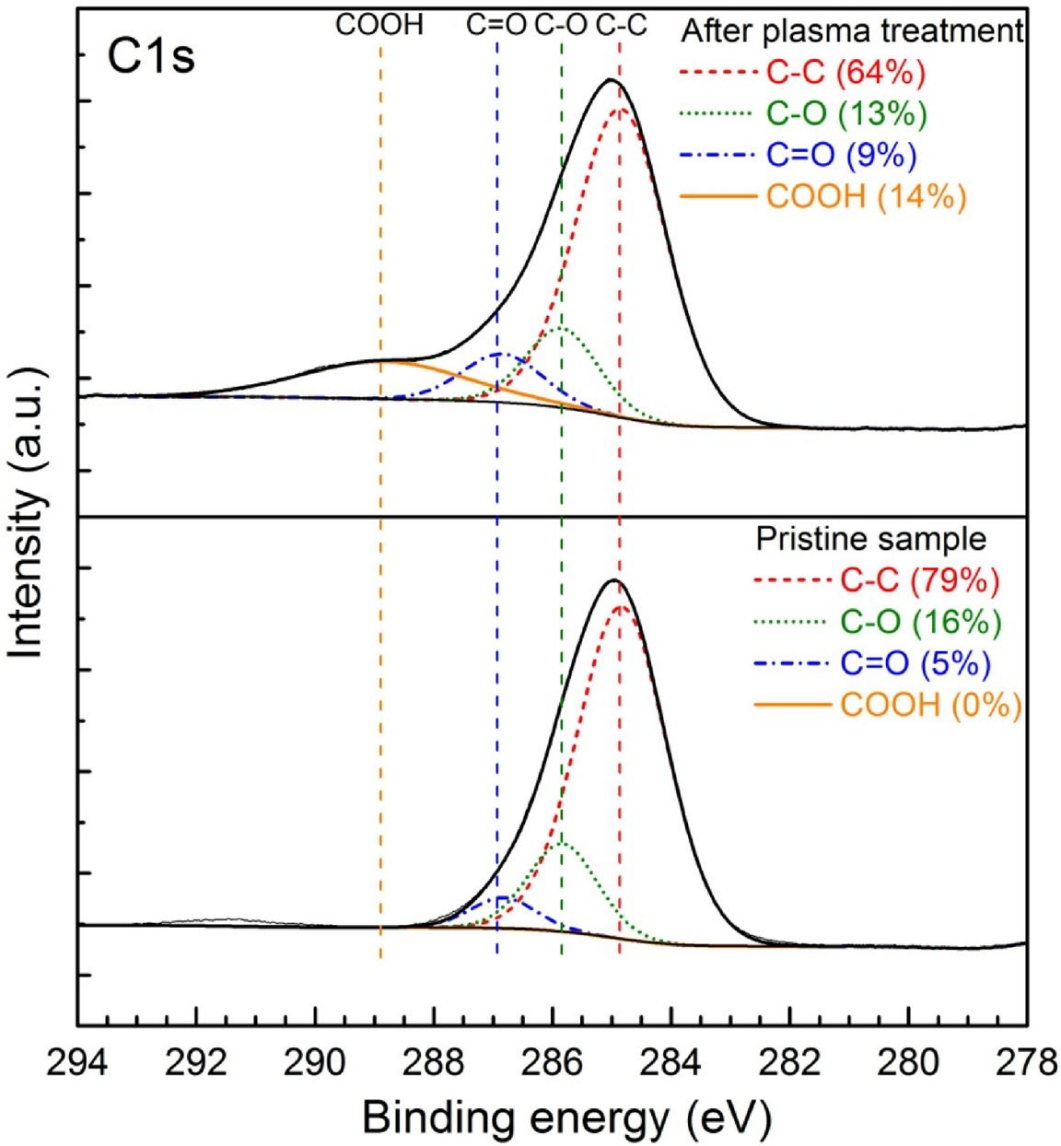
XPS analysis of C1s peak of PMMA polymer before and after plasma treatment measured in a binding energy interval of 294–278 eV. Argon flow rate 20 NL/min. Microwave power 460 W.

The more detailed study of the PMMA surface chemical composition before and after plasma treatment was proceed by using XPS. In such a way, in order to obtain the more valuable data on the functional groups the analysis of C1s and O1s peak was conducted. The high resolution XPS analysis of C1s peak of PMMA polymer before and after plasma treatment measured in a binding energy interval of 294–278 eV, for argon flow rate of 20 NL/min and absorbed microwave power of 460 W is presented in Fig. 12. As it can be seen the C1s peak of PMMA surface before plasma treatment was deconvoluted into three overlapped carbon-containing components. These were located approximately at 284.8 eV, 285.9 eV and 286.9 eV and were depicted to correspond to the following chemical bonds C–C (single carbon–carbon bond), C–O (carbon atoms singly bounded to oxygen) and C=O (carbon atoms with double bonds to oxygen atoms), respectively. The calculated percentage concentration of each above listed bonds of C1s spectra was equal to 79% for C–C bond, 16% for C–O bond and 5% in the case of C=O bond. As it can be further seen from Fig. 12 the XPS C1s spectra after plasma treatment varied. First of all, the changes in percentage of above mentioned carbon bonds were observed. Namely, the percentage concentration of C–C bond decreased from 79 to 64% after plasma treatment, the C–O bond decreased from 16 to 13% and the C=O bond increased from 5 to 9% after plasma treatment. Secondly, in the C1s spectra after plasma treatment a new peak located at 288.9 eV was observed. This peak was assigned to COOH carboxyl group. The binding energies of four above mentioned peaks of the XPS C1s spectra of plasma treated and plasma untreated PMMA sample were consistent with the results reported by other researchers^31,36,38,41^.

**Figure 13:**
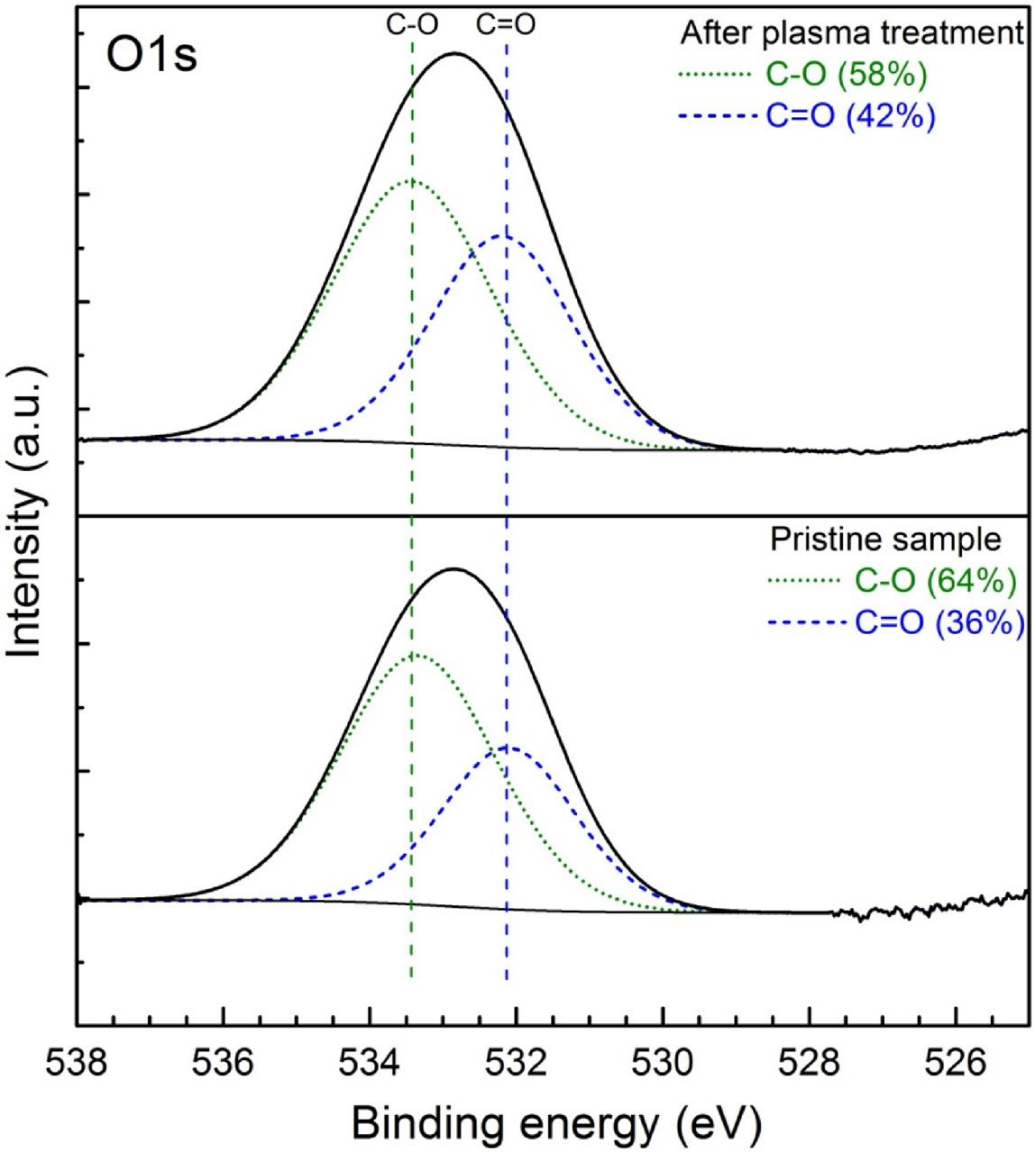
XPS analysis of O1s peak of PMMA polymer before and after plasma treatment measured in a binding energy interval of 538–527 eV. Argon flow rate 20 NL/min. Microwave power 460 W.

Figure 13 presents the XPS analysis of O1s peak of PMMA polymer before and after plasma treatment measured in a binding energy interval of 538–527 eV. The results correspond to an argon flow rate of 20 NL/min and an absorbed microwave power 460 W. As it can be seen, the deconvoluted spectra of pristine sample showed the presence of two peaks representing the oxygen components located at 532 eV and at 533.4 eV. These peaks are attributed to the single (C–O) and double (C=O) carbon–oxygen bond, respectively and their binding energies are in good agreement with other literature data^31,38^. The first peak percentage concentration was equal to 64%, while the second peak was equal to 36%. By comparing the XPS O1s spectra of PMMA surface before and after plasma treatment it may be concluded that no new functional groups were formed as a result of plasma modification, however the percentage of two oxygen-containing groups changed. The percentage concentration of C–O bond decreased from 64% for pristine sample to 58% for plasma treated sample, while the percentage concentration of C=O bond increased from 36 to 42%.

The XPS analysis of PMMA surface showed that the oxygen atoms content increased from 9% before plasma treatment to 29% after plasma treatment. At the same time the carbon atomic composition decreased from 91 to 71% for untreated and plasma treated PMMA surface, respectively. Thus, the oxygen-to-carbon ratio increased from about 0.1 for pristine sample to about 0.4 after plasma treatment. The increase in oxygen-to-carbon ratio indicate the increase of oxygen containing polar functional groups on the PMMA sample surface and increase in surface energy making the surface more hydrophilic. Such a relation is reflected in the presented in Figs. 5 and 6 results of the water contact angle measurements showing decrease of WCA after plasma treatment.

### Surface mechanical properties

**Figure 14:**
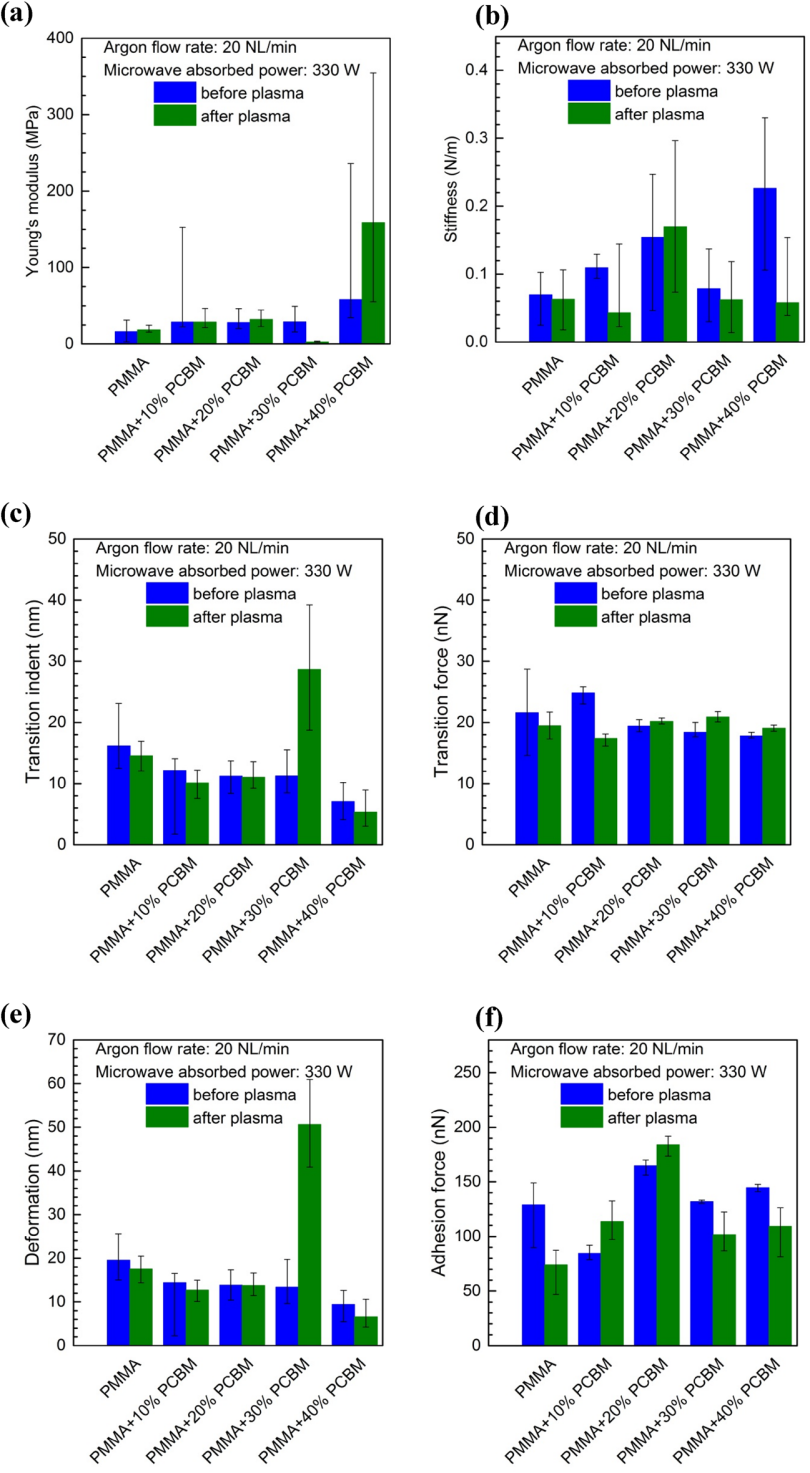
Effect of the plasma surface modification of PMMA polymer and PMMA + PC_61_BM composites on the surface mechanical properties parameters: (**a**) Young’s modulus; (**b**) stiffness; (**c**) transition indent; (**d**) transition force; (**e**) deformation; (**f**) adhesion force. Argon flow rate 20 NL/min. Microwave power 330 W.

Figure 14 shows the argon plasma treated PMMA surface mechanical properties parameters in comparison with the corresponding parameters for an untreated PMMA surface. Such surface mechanical properties parameters as Young’s modulus, stiffness, transition indent, transition force, deformation, and adhesion force are taken into account. The results presented in the figure correspond to an argon flow rate of 20 NL/min and microwave power of 330 W. It can be seen from the figure that some of the parameter changes resulting from plasma treatment are smaller, such as the case of the Young’s modulus of the PMMA material, while some of them are more significant, such as the case of the deformation of the PMMA + 30% PC_61_BM composite. Generally, the results presented in Fig. 14 indicate that the changes of each surface mechanical properties parameter are strongly dependent on the material it relates to. For example, in the case of the transition indent (Fig. 14c), where the changes, as it would seem, have a general tendency toward decreasing, or no significant changes being noted (PMMA + 20% PC_61_BM), for the PMMA + 30% PC_61_BM composite, a significant increase of its value was registered after plasma treatment. Otherwise, in the case of stiffness, the changes show in general a decrease of this value while the argon plasma was applied, however in the case of the PMMA + 20% PC_61_BM composite, a change is clearly in the opposite direction.

The analysis of mechanical parameters changes requires considering phenomena occurrence during the plasma processing. The polymer itself may change its structure due to high energy exposure as well as the rapid temperature increase and decrease. Therefore the various degradation mechanisms must be taken into account (i.e. degradation, depolymerization, thermal shock). As the consequence, among others, the morphological changes may be observed, as it was shown earlier. The mechanical response may in such a case be combined by the structural modification, some volume-scale defects introduction and roughness change. On top of that, also presence of PCBM may play a significant role, while its mechanical properties are very different than PMMA, and its presence in the matrix, may cause the stiffness increase when the certain level of molecules networking is achieved^69^. On the other hand, larger amount of the filler may cause the voids presence, therefore in addition to the matrix weakening, it may have large contribution to the mechanical resistance deterioration. Such complexity can be also considered in terms of the transition force and adhesion force, where the roughness change may induce the active contact tip-sample area. Also the PMMA degradation may exposure PCBM matrix, which can become more active in the tip-sample interaction, likely reducing the adhesion force if networked in solid bulky features. In specific conditions, one may consider creation of nano-anchoring features, creating relatively long-detachment distance, interpreted also as the adhesion force^70^. The impact of PCBM amount may have modify deformation depth, as the resistance provided by the filler may cause more effective elastic deformation instead inelastic deformation, therefore this particular parameter can in general decrease while the filler amount is higher. This parameter shows similar changes as the transition indent, therefore one can apply similar interpretation to those results. We would like to underline, that further research should allow us to explain the roles of certain mechanism we mentioned above, and likely, identify also more phenomena.

In the paper^71^ concerning polypropylene surface treatment using cold air plasma the relationship between wettability and adhesion was experimentally investigated. As a conclusion authors claimed that that adhesion and wettability are two related properties. According to this, such a kind of relationship seems to be observed also in the case of our experimental investigations. By comparison the results presented in Figs. 6 and 12 it can be noticed that in general the water contact angle and the adhesion force follow the same trend (increase or decrease) after plasma treatment. This means an increase or decrease in the values of both parameters at the same time after plasma treatment. However, this conclusion seems to be very general and requires further investigations.

## Conclusions

An atmospheric pressure microwave (2.45 GHz) argon plasma source was used to modify the surface of the PMMA polymer, and PMMA + PC_61_BM composite materials. Its major merit (apart from the electrodeless operation) was the unique shape of the generated plasma, having the form of plasma sheet consisting of numerous fluctuating argon filaments protruding from the quartz box, and thus enabling surface treatment. After the exposure of the tested materials to the argon plasma at various argon flow rates and microwave powers, the plasma modified materials’ surface parameters were evaluated using water contact angle goniometry, atomic force microscopy, and attenuated total reflectance Fourier transform infrared spectroscopy.

The obtained results showed that the PMMA polymer’s surface can be altered into hydrophilic by treating it by a single argon plasma scan at sample transfer velocity of 10 mm/s. However, the reduction in water contact angle due to the plasma treatment is more significant in the case of the PMMA polymer, and the PMMA + 40% PC_61_BM composite than in the case of other PMMA + PC_61_BM composites. The best result reported herein of water contact angle decrease was from 83° to 29.7° for the PMMA material. Ageing studies of the PMMA plasma-modified surface showed the long term stability (100 h) of the water contact angle of modified PMMA samples stored in a non controlled environment (ambient air conditions).

The study of the role of the number of plasma scans on the surface roughness parameters showed that in the case of the PMMA polymer, as well as in the case of the PMMA + PC_61_BM composites, it is an irregular dependence. This means that it is not possible to determine any even general trend of influences of the number of plasma scans on the particular surface roughness parameters.

The comparison of the ATR-FTIR surface spectra of a PMMA pristine sample and an argon plasma-modified PMMA sample did not reveal any significant changes, implying that no new functional groups were formed on the sample surface. Only small differences in the intensities of certain spectra peaks were registered. The XPS study revealed in more detail the changes in surface composition and chemical bonding of the PMMA sample after plasma treatment indicating at the same time the increase of oxygen to carbon ratio from 0.1 for pristine sample to 0.4 for plasma treated sample.

As it is well known, the hydrophilic-hydrophobic properties of the polymer's surface are determined by the surface chemical composition and the surface morphological properties^26,35,40^. Thus, the polymer’s surface wettability is not only affected by the e.g. existence of polar hydrophilic groups but also by the surface roughness. The more detailed study on the correlation of the surface roughness with the surface wettability (WCA) can be found in the literature^72–74^. Thus, it may be concluded that in our case the changes in wettability of the plasma modified samples’ surfaces are a result of changes in surface morphology (roughness) as well as in surface chemistry.
